# Thymic Stromal Lymphopoietin Enhances Th2/Th22 and Reduces IL-17A in Protease-Allergen-Induced Airways Inflammation

**DOI:** 10.1155/2013/971036

**Published:** 2013-02-07

**Authors:** Dieudonnée Togbe, Louis Fauconnier, Fahima Madouri, Tiffany Marchiol, Pauline Chenuet, Nathalie Rouxel, Aurélie Ledru, François Erard, Valerie Quesniaux, Bernhard Ryffel

**Affiliations:** ^1^Artimmune SAS, 13 avenue Buffon, 45071 Orléans, France; ^2^INEM, CNRS, UMR 7355, University of Orléans, 3B rue de la Férollerie, 45071 Orléans Cedex 2, France; ^3^Institute of Infectious Disease and Molecular Medicine, University of Cape Town, 7701 Rondebosch, South Africa

## Abstract

*Background*. Thymic stromal lymphopoietin (TSLP) is induced in allergic skin and lung inflammation in man and mice. *Methods*. Allergic lung inflammation induced by two proteases allergens HDM and papain and a classical allergen ovalbumin was evaluated *in vivo* in mice deficient for TSLPR. Eosinophil recruitment, Th2 and Th17 cytokine and chemokine levels were determined in bronchoalveolar lavage fluid, lung homogenates and lung mononuclear cells *ex vivo*. *Results*. Here we report that mice challenged with house dust mite extract or papain in the absence of TSLPR have a drastic reduction of allergic inflammation with diminished eosinophil recruitment in BAL and lung and reduced mucus overproduction. TSLPR deficient DCs displayed diminished OVA antigen uptake and reduced capacity to activate antigen specific T cells. TSLPR deficient mice had diminished proinflammatory IL-1**β**, IL-13, and IL-33 chemokines production, while IL-17A, IL-12p40 and IL-10 were increased. Together with impaired Th2 cytokines, IL-17A expressing TCR**β** ^+^ T cells were increased, while IL-22 expressing CD4^+^ T cells were diminished in the lung. *Conclusion*. Therefore, TSLPR signaling is required for the development of both Th2 and Th22 responses and may restrain IL-17A. TSLP may mediate its effects in part by increasing allergen uptake and processing by DCs resulting in an exacerbated asthma.

## 1. Introduction

The allergic inflammatory response is characterized by a predominant Th2-cell pathway, which is initiated by the uptake of allergens by professional antigen presenting cells (APCs) that present selected peptides on MHC class II molecules to naive T cells, together with isotype switching of B cells to generate IgE antibodies specific for common environmental allergens [1]. The cytokines associated with Th2 response are IL-4, IL-5, IL-9, IL-13, and IL-33 [2, 3]. Thymic stromal lymphopoietin (TSLP) was first identified as a growth-promoting factor produced by mouse thymic stromal cells that supported the development of immature B cells to the B220^+^/IgM^+^ stage [4]. TSLP is a type I cytokine that acts via the heteromeric receptor consisting of IL-7R*α* and a TSLP-specific subunit, TSLP receptor (TSLPR) [5, 6] signaling via JAK1 and JAK2 to mediate the activation of STAT5A and STAT5B [7]. TSLPR has homology to the common cytokine receptor *γ*-chain, *γ*_c_, a component of the receptors for IL-2, IL-4, IL-7, IL-9, IL-15, and IL-21 [8]. 

TSLP is expressed by a range of cell types, including epithelial cells, fibroblasts, keratinocytes, mast cells, protease-activated basophils, human CD68^+^ macrophages, and myeloid DCs (mDCs) whereas it is not produced by other lympho-hematopoietic cells, including neutrophils, B cells, T cells, monocytes, plasmacytoid DCs (pDCs), and endothelial cells [9, 10]. 

TSLP acts on many cell types including dendritic cells (DCs) [9], T cells [11, 12], mast cells [13], NKT cells [14] and eosinophils [15]. Furthermore, TSLP may act via DCs to regulate the activation, differentiation, and homeostasis of T cells [16], but it also has direct effects on T cells, promoting their survival and proliferation in response to TCR activation [17]. 

TSLP has been implicated in the development of asthma [11, 18], atopic dermatitis, inflammatory arthritis, and other inflammatory disease conditions [16, 19]. Interestingly, TSLPR knockout (KO) mice have a defective allergic inflammatory response to OVA in the lung, but this can be reversed by adoptive transfer of wild-type (WT) CD4^+^ T cells [11], underscoring a key role for the action of TSLP on these cells. Moreover, TSLP induces Th-2 attracting chemokines and primes naives Th-2 cells to produce IL-4, IL-5 and IL-13, and TNF*α* and inhibits Th-1 differentiation [11].

It was demonstrated recently that papain activated basophils or HDM activated airways stromal cells also produce TSLP and thus may be important in the initiation of Th2 responses [20, 21]. Moreover, when lung cells were sorted into epithelial cells or DCs, TSLP mRNA was expressed by the epithelial cells and by the DCs [20].

Here we extended our investigation on the role of TSLP in allergic asthma using clinically relevant protease type allergens, house dust mite (HDM) extract and papain. We demonstrate defective DC help for T cells and diminished Th2 and Th22 and enhanced Th17 responses with diminished allergic airways inflammation in TSLPR deficient mice.

## 2. Material and Methods

### 2.1. Materials

*O*-phenylenediamine, 3-amino-1,2,4-triazole, horseradish peroxidase, and BSA grade V were obtained from Sigma Chemical Company (St. Louis, MO). The antibodies used for FACS analysis, FITC-anti CD3e (clone 145-2C11), PE-anti-IL-17A (clone TC11-18H10), PerCP-anti-CD4 (clone RMA-5), biotin-anti CD8*α* (clone 53-6.7), biotin-anti TCR*β* (clone H57-597) and Isotype-matched controls were purchased from Pharmingen (San Diego, CA). APC-anti-IL-17F (clone eBio18F10), FITC-anti TCR*γδ* (clone GL3) and PerCPeF710-anti-IL-22 (clone 1H8PWSR) antibody were purchased from eBioscience. 

### 2.2. Mice

C57BL/6 wild type mice and TSLP-R^−/−^ were bred in our specific pathogen free animal facility at CNRS (Orleans, France). TSLPR^−/−^ mice (on C57BL/6 genetic background) were from the laboratory of molecular immunology, National Heart, Lung and Blood Institute (Dr W. Leonard, Bethesda, USA) [11]. Mice were maintained in a temperature-controlled (23°C) facility with a strict 12 h light/dark cycle and were given free access to food and water. The experiments were performed with gender-matched mice aged 8–10 weeks. All protocols complied with the French Government's ethical and animal experiment regulations.

### 2.3. Allergic Airway Inflammation Induction

For HDM model, mice were immunized by intranasal route at days 0 and 7 with 25 *μ*g of HDM extracts (ALK Abello, Danemark). On day 14, 15 and 16, mice were challenged by intranasal route with 5 *μ*g of HDM extracts.

For OVA model, mice were sensitized subcutaneously twice at days 0 and 7 with 200 *μ*L saline containing 10 *μ*g Ovalbumin (OVA, grade V, Sigma) without aluminum adjuvant. One week after the second sensitization, mice were challenged 3 times by intranasal routes (on day 14, 15 and 16) with 40 *μ*L of saline containing 10 *μ*g OVA. Control mice were challenged with saline alone. Mice were killed with CO_2_ inhalation after the last challenge via a tracheal canula, lungs were washed 4 times with 0.5 mL of saline solution (see below Bronchoalveolar lavage).

For protease allergen papain model, mice were anesthetized by isoflurane inhalation, followed by intranasal administration of papain (25 *μ*g, Calbiochem) in 40 *μ*L of saline on days 0–2 as described [22]. Mice were killed on day 3 and BAL was performed. After bronchoalveolar lavage, lungs were perfused via heart puncture with ISOTON II Acid free balanced electrolyte solution (Beckman Coulter, Krefeld, Germany). Half of the lung was stored at −80°C for EPO enzyme, cytokines, and chemokines analysis and the other half was fixed overnight in buffered 4% formaldehyde solution for histology analysis. BAL fluid was analyzed for cell composition and cytokine concentrations. Experiments were performed at least twice using groups of 8 animals.

### 2.4. Bronchoalveolar Lavage (BAL)

Bronchoalveolar lavages (BAL) were performed by washing the lungs 4 times with 0.5 mL of saline solution at room temperature. BAL cells were sedimented by centrifugation at 400 ×g for 10 min at 4°C. The supernatant (cell-free BAL fluid) was stored at −20°C for cytokine analysis. An aliquot of the cell pellets was stained with Trypan blue solution, counted, and 100,000 cells centrifuged on microscopic slides (cytospin at 1000 rpm for 10 min, at RT). Air-dried preparations were fixed and stained with Diff-Quik (Merz & Dade A.G., Dudingen, Switzerland). Differential counts were made under oil immersion microscopy at ×80 magnification. One hundred cells were counted twice for the determination of the relative percentage of each cell type present in the BAL.

### 2.5. Lung Histology

The organs were fixed in 4% buffered formaldehyde overnight and embedded in paraffin. Lung sections of 3 *μ*m were stained with periodic acid Schiff reagent (PAS) and examined with a Leica microscope (×20 magnification). Peribronchial infiltrates and mucus hypersecretion were assessed by a semi-quantitative score (0–3) by two observers independently.

### 2.6. Pulmonary Eosinophil Peroxidase (EPO) Activity

EPO activity was determined in order to estimate the recruitment of eosinophils to the lung parenchyma. After BAL and perfusion, lungs were excised, stored frozen at −80°C or directly homogenized for 30 seconds in 1 mL of 0.05 M Tris/HCl buffer pH 8.0 using a Polytron (Kinematic AG, Luzern, Switzerland). The homogenate was centrifuged for 15 min at 4°C at 10,000 ×g. EPO activity in the supernatant was determined as estimated from the oxidation of *O*-phenylenediamine (OPD) by EPO in the presence of hydrogen peroxide (H_2_O_2_) using the protocol by Van Oosterhout and colleagues [23]. The substrate solution consisted of 10 mM OPD in 0.05 M Tris/HCl-buffer (pH = 8) and 4 mM H_2_O_2_ (BDH, Poole, UK). Substrate solution was added to samples in a 96-wells microplate (Greiner) and incubated at 37°C for 30 min. Duplicate incubations were carried out in the absence and presence of the EPO inhibitor 3-amino-1,2,4-triazole (AMT, 2 mmol/L). The absorbance was then measured at 490 nm (Flow Labs, Irvine, UK). Results are expressed as OD 490 nm and were corrected for the activity of other peroxidases, which were not inhibited by AMT.

### 2.7. Quantification of Cytokines

The lungs were homogenized for 30 s using a Polytron (Kinematic AG, Luzern, Switzerland) and the cell debris were eliminated by centrifugation at 10,000 ×g for 15 min. IL-1*β*, IL-13, IL-33, TSLP, CCL11, CCL17 CCL22, and CCL24 concentrations in BAL or lung homogenate supernatants were determined by enzyme-linked immunosorbent assay (ELISA), using commercial kits from R&D (Abingdon, UK). IL-10, IL-12p40, IL-17A, and IFN*γ* were determined by Bio-Plex mouse Cytokine Group I 23-Plex on MagPix (Luminex, Bio Rad) according to the manufacturers' instructions.

### 2.8. Bone Marrow Derived Dendritic Cells (BMDCs) Culture

Murine bone marrow cells were isolated from femurs of wild type and TSLPR^−/−^ mice and differentiated into myeloid dendritic cells (DCs) by culturing at 1 × 10^6^ cells/mL for 10 days in RPMI medium supplemented with 10% FCS (Hyclone), non-essential amino-acids, 0.05 *μ*g/mL asparagine, MEM vitamins, sodium pyruvate, gentamycin (2 *μ*M, Invitrogen), penicillin (100 U/mL, Gibco, Invitrogen), 10 mg/mL streptomycin, 2-mercaptoethanol 50 *μ*M and 4% J558L cell-conditioned medium as a source of GM-CSF (change medium on days 3, 6, and 8). Dendritic cells were treated with 100 *μ*g/mL OVA-FITC (Molecular probes, France) for 2 h and analyzed by FACS. The data are given as the mean fluorescence intensity (MFI).

### 2.9. *In Vitro* T-Cell Proliferation

 Lymph node CD4^+^ T cells were purified from OT2 mice by magnetic cell Sorting (Dynal, Invitrogen). CD4^+^ T cells (10^5^ cells) were co-cultured with 10^4^ WT or TSLPR^−/−^ dendritic cells preloaded with OVA peptide (10 *μ*g/mL, 2 h). T cell proliferation was assessed by CFSE staining (0.5 *μ*M; Molecular probes, Invitrogen).

### 2.10. Lung Mononuclear Cells Isolation

Lung mononuclear cells were isolated from mice 24 h after the last challenge as described [24]. Briefly the aorta and the inferior vena cava were sectioned and the lungs were perfused with saline. The lobes of the lungs were sliced into small cubes and then incubated for 20 min in 1 mL RPMI 1640 solution containing DNase I (1 mg/mL) and collagenase IV (2 mg/mL) (Sigma-Aldrich). Lung mononuclear cells were separated by centrifugation on Percoll (Amersham Biosciences) gradients (37%). Isolated lung mononuclear single cells were plated in round bottom 96-well plates (2 × 10^6^/mL) and restimulated 4 h *in vitro* with phorbol 12-myristate 13-acetate (PMA) (50 ng/mL) and ionomycin (750 ng/mL; both from Sigma-Aldrich) in complete medium (IMDM supplemented with 5% (vol/vol) FCS, L-glutamine (2 *μ*M), penicillin (100 U/mL), streptomycine (100 *μ*g/mL), and *β*-mercaptoethanol (50 nM) all from Invitrogen).

### 2.11. Flow Cytometry Analysis on Lung Mononuclear Cells

Cell suspensions from lung were restimulated *in vitro* for 4 h in complete medium with PMA (50 ng/mL) and ionomycin (750 ng/mL; both from Sigma-Aldrich) in presence of Brefeldine A (GolgiPlug, BD Biosciences, France). To prevent a specific binding to FcR, 2.4.G2 blocking purified antibody was used. After 4 h, cells were stained with the following monoclonal antibodies, FITC-labeled TCR*γδ*, biotin-labeled TCR*β*,-V450-labeled CD4, and APC-Cy7-labeled CD8*α*. After washing, cells were permeabilized for 20 min with cytofix/cytoperm kit (BD Biosciences, France) and stained with APC labeled IL-5, PE-labeled IL-17A and PerCPeFluor710—labeled IL-22. Samples were analyzed on a BD CANTO II flow cytometer. Fluorescence data were acquired by using DIVA software (BD Bioscience, France) and analyzed using FlowJO software (Treestar).

### 2.12. Statistical Analysis

The data are presented as the mean ± SEM with *n* = 6–8 animals per condition. The significance of differences between two groups was determined by one way ANOVA (non parametric test) using Prism software. Statistical significance was reported if *P* < 0.05 was achieved, *P ≤ 0.05; **P ≤ 0.01; ***P ≤ 0.001.

## 3. Results

### 3.1. Reduced Eosinophil Recruitment in Response to House Dust Mite Allergen

**Figure 1:**
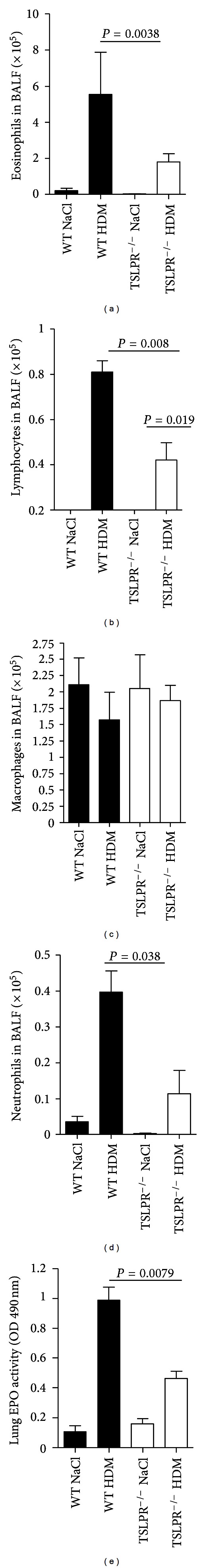
Reduced eosinophils influx in TSLPR^−/−^ mice during HDM induced allergic asthma lung inflammation. HDM sensitized WT and TSLPR^−/−^ mice (C57BL/6 background) were challenged three times with HDM inhalation. 24 h after the third challenge, the number of eosinophils (a), the lymphocytes (b), macrophages (c), and neutrophils (d) were determined in BALF and EPO activity in lung tissue (e). These experiments were performed twice (*n* = 8 mice per group). One representative experiment is shown. Values are the mean ± SEM of 8 mice per group.

HDM is a major source of allergens in allergic patients and cause allergic airway inflammation resembling human asthma in mice by facilitating barrier disruption, inflammation, and allergen sensitization of the airways through TLR4-dependent innate and acquired immunity [20, 25, 26]. Previous studies demonstrated reduced allergic response to ovalbumin in TSLPR^−/−^ mice [11], here it was asked whether the inflammatory response to the clinically relevant allergen HDM is dependent on TSLPR signaling. Mice were immunized twice and challenged on days 14, 15 and 16 by intranasal instillation of HDM and the BAL fluid and lung tissues were analyzed on day 17. Eosinophil, neutrophil and lymphocyte influx in the alveolar space were significantly reduced in TSLPR^−/−^ mice while macrophages were unchanged (Figures 1(a)–1(d)). Furthermore eosinophil peroxidase activity (EPO) was also reduced in the lung tissue (Figure 1(e)). 

**Figure 2:**
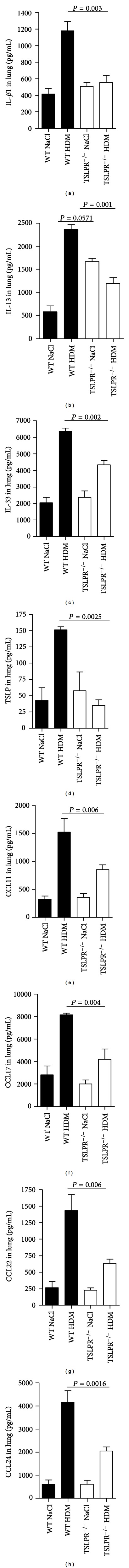
Decreased pulmonary Th2 cytokine and chemokine responses in TSLPR^−/−^ mice in response to HDM. Mice were immunized and challenged with HDM as before. IL-1*β*, IL-13, IL-33, TSLP, CCL11 (Eotaxin-1), CCL17 (TARC), CCL22 (MDC), and CCL24 (Eotaxin-2) were measured in the lung homogenate by ELISA (a–h) from HDM treated WT and TSLPR^−/−^ mice at 24 h after the third challenge. These experiments were performed twice (*n* = 8 mice per group). One representative experiment is shown. Values are the mean ± SEM of 8 mice per group. **P ≤ 0.01; ***P ≤ 0.001.

IL-13 and IL-33 are known to drive eosinophil maturation and infiltration, mucus production and bronchial hyperreactivity. To investigate whether TSLP signaling disruption could affect cytokine production, we induced local airway allergic inflammation with HDM on TSLPR^−/−^ mice. The analysis of cytokines in lung homogenate revealed a drastic reduction of IL-1*β*, IL-13, and IL-33 in the absence of TSLPR signaling suggesting a significant reduction of Th2 associated local response to HDM (Figures 2(a)–2(c)). Furthermore the chemokines CCL11, CCL17, CCL22, and CCL24 were significantly reduced upon HDM allergen exposure (Figures 2(e)–2(g)) underscoring a defect of eosinophil recruitment in the absence of TSLPR. The absence of TSLP upregulation in TSLPR^−/−^ mice suggests an autocrine loop of TSLP production in the lung (Figure 2(d)). Thus, the data extend the notion of a critical role of TSLPR to generate a Th2 cytokine/chemokine milieu.

### 3.2. Eosinophilic Lung Inflammation Depends on TSLPR

**Figure 3:**
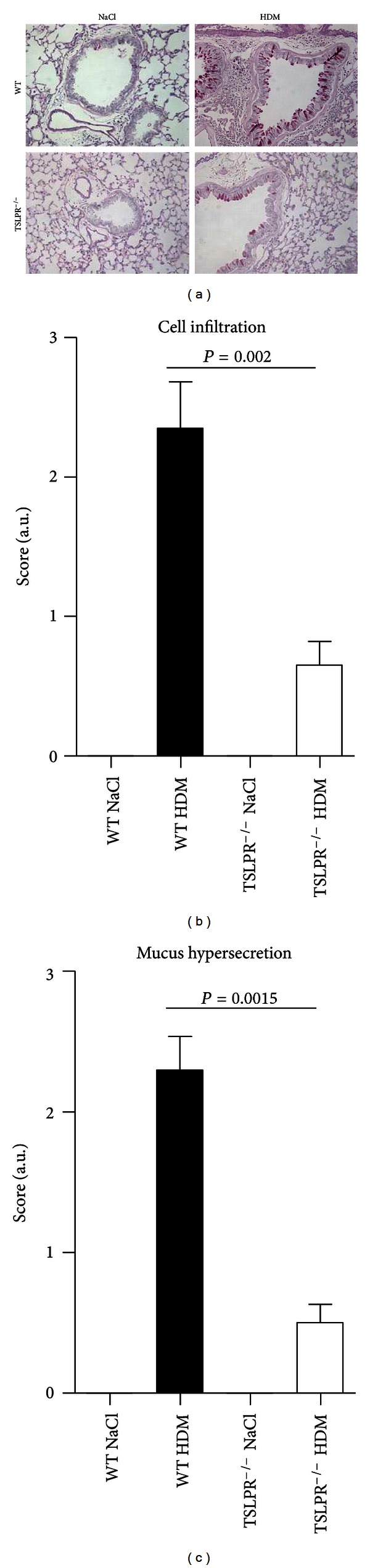
Reduced lung inflammation in TSLPR^−/−^ mice in response to HDM. The formalin-fixed lung sections were stained with periodic acid Schiff reagent (PAS) to visualize mucus (a). Magnification ×20. Representative sections from WT saline control, HDM treated WT, and TSLPR^−/−^ mice are shown. A semi-quantitative histological assessment of cell infiltration and mucus hypersecretion was performed by two independent observers (b). A scale from 0 to 3 is given on the axis. These experiments were performed twice (*n* = 8 mice per group). One representative experiment is shown. Values are the mean ± SEM of 8 mice per group.

In view of an important role of TSLPR in HDM induced allergic inflammation we examined the lung tissue at day 17. HDM immunized and challenged WT mice developed a robust inflammation with abundant eosinophils and mucus production in the bronchial epithelial cells (Figures 3(a) and 3(b)). By contrast eosinophilic inflammation and mucus production was largely abrogated in the absence of TSLPR. Therefore, TSLPR signaling is required for an allergic inflammatory response to HDM. 

### 3.3. TSLPR Is Required for the Development of Innate Type Airway Inflammation Induced by Papain

**Figure 4:**
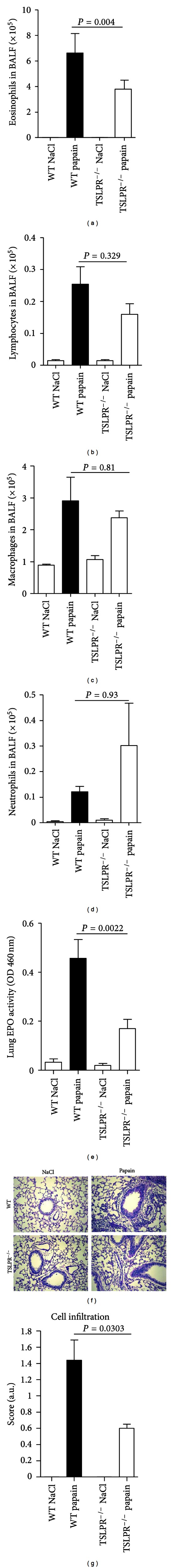
TSLPR is essential for the development of innate type airway inflammation induced by papain. Mice were exposed daily to 25 *μ*g papain for 3 days and analyzed 24 after the last intranasal instillation. The number of eosinophils (a), lymphocytes (b), macrophages (c), and neutrophils (d) were determined in BALF as well as EPO activity in lung tissue (e) was determined 24 h after the last papain or saline control administration in wild-type (WT) and TSLPR^−/−^ mice. Lung sections stained with Hematoxylin-Eosin (HE) (20x magnification) and score of the severity of inflammation and mucus production at 24 h after the last papain or PBS inhalation are shown (f-g). These experiments were performed twice (*n* = 8 mice per group). One representative experiment is shown. Values are the mean ± SEM of 8 mice per group.

**Figure 5:**
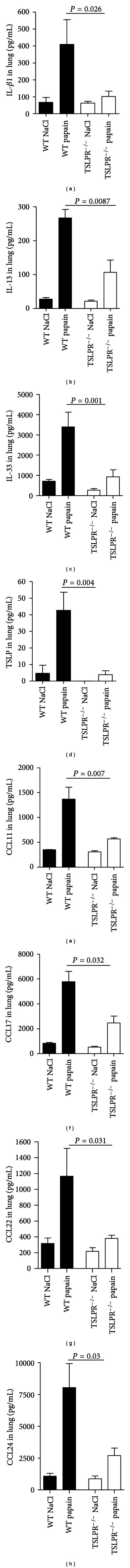
Diminished cytokine and chemokine expression in TSLPR^−/−^ mice in innate type of lung inflammation induced by papain. IL-1*β*, IL-13, IL-33, TSLP, CCL11 (Eotaxin-1), CCL17 (TARC), CCL22 (MDC), and CCL24 (Eotaxin-2) in the lung homogenate after papain exposure were determined by ELISA (a–h). These experiments were performed twice (*n* = 8 mice per group). One representative experiment is shown. Values are the mean ± SEM of 8 mice per group.

Papain, a cysteine protease, was shown to preferentially induce an IgG1 response and results in mast cell degranulation, both features typical of an allergic reaction [27]. It has recently been shown that the protease papain could induce asthma like symptoms in RAG-deficient mice [22, 28]. This effect is mediated by innate lymphocytes also known as natural helper cells or nuocytes cells. Therefore we tested whether TSLPR signaling is involved in papain induced lung inflammation. Intranasal administration of papain into TSLPR^−/−^ mice showed a dramatic decrease of eosinophils in BAL fluid and eosinophil peroxidase activity in the lung, while lymphocyte, macrophage and neutrophil recruitment into BALF was not affected (Figures 4(a)–4(e)). Histological examination revealed that lung inflammation in papain treated TSLPR^−/−^ mice was substantially reduced than in WT mice (Figures 4(f) and 4(g)). Interestingly, papain induced Th2 and inflammatory cytokines such as IL-1*β*, IL-13, IL-33, and TSLP (Figures 5(a)–5(d)) were profoundly impaired in TSLPR^−/−^ mice as well as the chemokines CCL11, CCL17, CCL22, and CCL24 (Figures 5(e)–5(h)). Therefore our data demonstrate that TSLP is required in papain induced eosinophil recruitment, pulmonary inflammation and Th2 cytokine production.

### 3.4. Reduced Antigen Uptake in DCs and T Cell Response in the Absence of TSLPR

**Figure 6:**
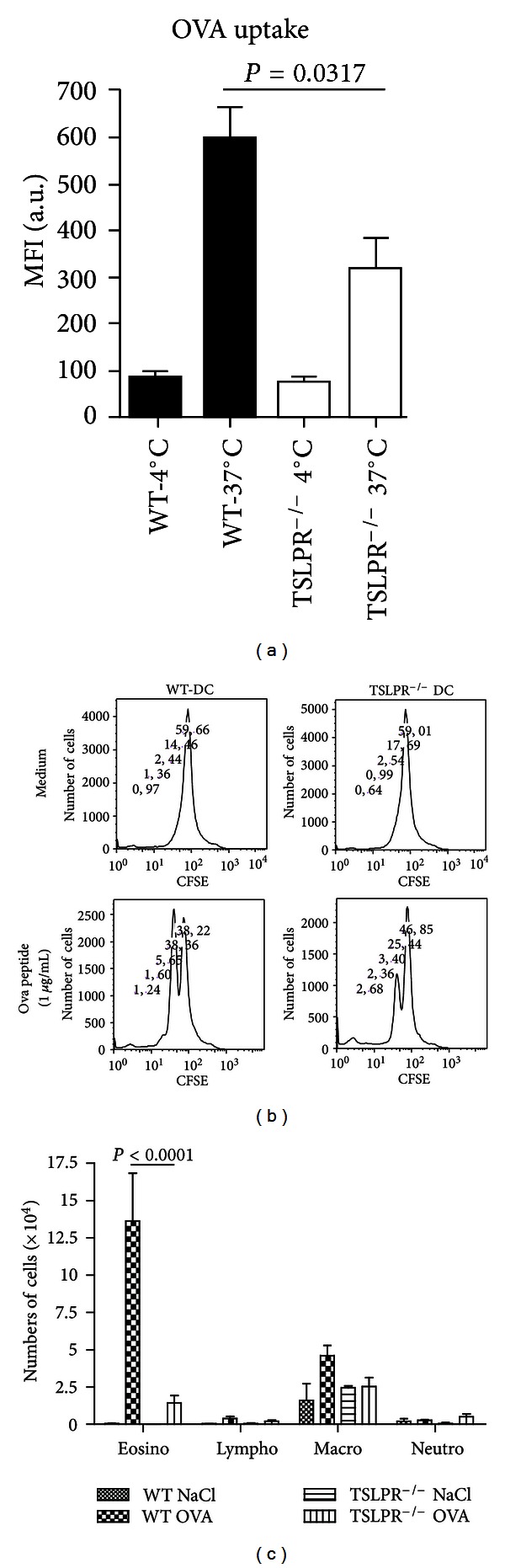
Reduced antigen uptake and eosinophils influx in TSLPR^−/−^ mice upon OVA induced allergic asthma model. Dendritic cells were differentiated *in vitro* from naive bone marrow derived cells. Uptake of OVA-FITC by dendritic cells after 2 h was analyzed by FACS (100 *μ*g/mL). The data are given as the mean fluorescence intensity (MFI). OVA peptide specific T cell proliferation was assessed by coculture of DC from WT or TSLPR^−/−^ mice loaded with OVA peptide (10 *μ*g/mL) with CFSE labelled CD4^+^ OT2 T cells (b). Critical role of TSLPR signalling for allergic inflammatory cell recruitment in BALF in OVA immunized and challenged mice (c). OVA sensitized WT and TSLPR^−/−^ mice were challenged three times with OVA instillation. 24 h after the third challenge, eosinophil, lymphocyte, macrophage, and neutrophil recruitment in BAL was determined. These experiments were performed twice (*n* = 8 mice per group). One representative experiment is shown. Values are the mean ± SEM. of 8 mice per group.

In view of the data suggesting a critical and autocrine effect of TSLP via TSLPR expressing DC [29], we investigated antigen uptake by DC in presence or absence of TSLPR and the subsequent T cell response. For this investigation we used OVA as antigen in order to use peptide specific OT2 T cells since HDM TCR transgenic are not established to assess T cell proliferation. We found reduced uptake of FITC-labeled OVA by TSLPR^−/−^ DC (Figure 6(a)). Furthermore the proliferation of the OVA peptide specific OT2 T cells in response to OVA peptide pulsed TSLPR^−/−^ DC was reduced as compared to WT DC (Figure 6(b)). Finally we verified the previous data that eosinophil recruitment in the bronchoalveolar space in OVA immunized and challenged TSLPR^−/−^ mice (Figure 6(c)). Therefore, TSLPR signaling in DCs is required for antigen uptake and presentation to activate CD4 T cells, consistent with a recent report demonstrating TSLP production and response by DC [29]. Since Th17 cell differentiation [15] and allergic lung inflammation [30, 31] contribute to allergic inflammation, we asked whether TSLPR signaling may contribute to Th17 cell response. 

### 3.5. Enhanced TCR*β* ^+^IL-17A^+^ and Reduced CD4^+^IL-22^+^ T Cells Recruitment in the Absence of TSLPR 

**Figure 7:**
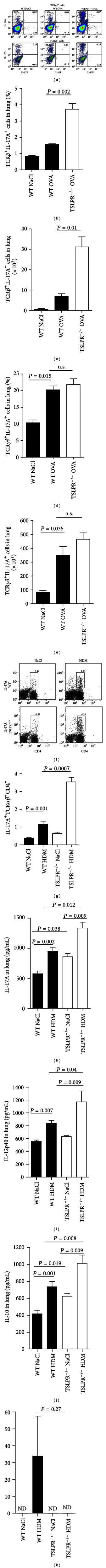
Increased pulmonary IL-17A^+^ cell populations in the absence of TSLPR. Lung mononuclear cells from OVA or HDM sensitized and challenged WT and TSLPR^−/−^ mice were isolated and restimulated for 4 h with PMA (50 ng/mL) and ionomycin (750 ng/mL) followed by membrane staining of TCR*αβ* and TCR*γδ*. Representative dot plot, frequency and absolute numbers of IL-17A^+^ producing T cells gated either on TCR*αβ* ^+^ or TCR*γδ* ^+^ T cell populations (a–e) are shown for OVA model. Representative dot plot and the frequency of IL-17A^+^ producing cells gated on TCR*αβ* ^+^ T cell populations (f, g), IL-17A (h), IL-12p40 (i), IL-10 (j) and IFN*γ* (k) levels in lung supernatant from HDM treated WT and TSLPR^−/−^ mice are shown. Values are the mean ± SEM. of 6–8 mice per group.

We reported that IL-17A is required [32, 33] and IL-22 reduces the allergic responses [34, 35]. Therefore, we asked whether IL-17A expression in T cells from the lung of OVA or HDM immunized and challenged mice is altered. Pulmonary IL-17A^+^TCR*αβ* ^+^ and IL-17A^+^TCR*γδ* ^+^ cells were significantly increased in WT mice, but the recruitment of the TCR*αβ* ^+^IL-17A^+^ cells augmented much more in the absence of TSLPR (Figures 7(a)–7(h)). 

To address the mechanisms underlying increased IL-17A level and diminished Th2 response in TSLPR^−/−^ mice, we examined the levels of cytokines shown to promote IL-17A level and regulatory T cells in the airways. Analysis of IL-12p40, IFN*γ*, and IL-10 in the lung homogenate revealed that IL-12p40 and IL-10 levels were increased in TSLPR^−/−^ mice treated with HDM, while IFN*γ* level was not detectable (Figures 7(i)–7(h)). 

**Figure 8:**
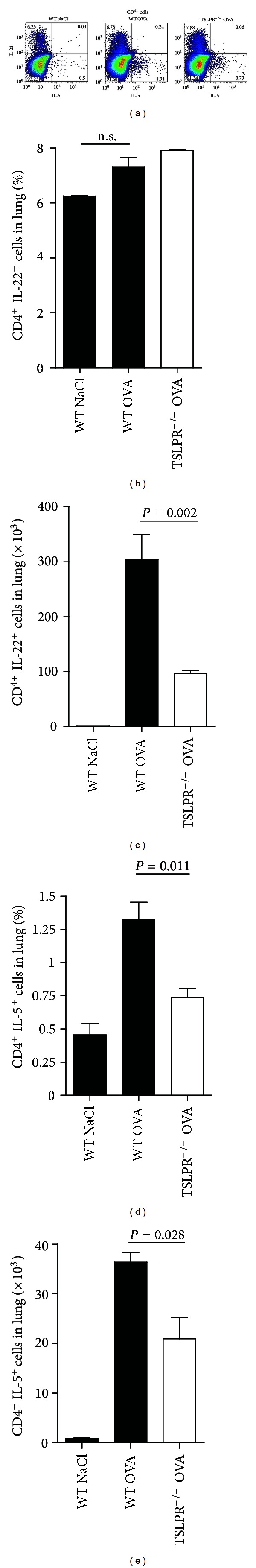
Reduced pulmonary IL-5^+^ and IL-22^+^ cell populations in the absence of TSLPR. Lung mononuclear cells from OVA sensitized and challenged WT and TSLPR^−/−^ mice were isolated and restimulated for 4 h with PMA (50 ng/mL) and ionomycin (750 ng/mL) followed by extracellular staining of TCR*γδ* and CD4. Representative dot plot, the frequency and the absolute numbers of IL-5^+^ and IL-22^+^ producing cells gated on TCR*αβ* ^+^CD4^+^ T cell populations (a–e) are shown. Values are the mean ± SEM of 6 mice per group.

Since we previously reported a cross-regulation of IL-22 and IL-17 [34], we investigated the expression of IL-22 a pulmonary T cells in the absence of TSLPR. We found a significant reduction of total CD4^+^IL-22^+^ T cells from the lung of TSLPR^−/−^ mice, while the total lung CD4^+^IL-5^+^T cells were not significantly reduced (Figures 8(a) and 8(b)). 

Therefore, TSLPR signaling is involved in the balance of Th17/Th22, in favor of the development of the Th22 subset, suggesting that physiologically TSLP dampens IL-17A and enhances IL-22 production. Based on our previous work showing a reciprocal role of IL-17A and IL-22 [34], the altered balance of IL-17A and IL-22 may contribute to the diminished allergic lung response.

## 4. Discussion

Several studies linked TSLP to lung inflammation and helminth infection [18, 19, 36], although the role of TSLP in airway inflammation using clinically relevant protease allergens HDM and papain have not been yet addressed.

Here we demonstrate that the allergic inflammatory response to protease allergens HDM or papain is dependent on TSLPR signaling. Proteases are important components of many allergens and thought not only to disrupt mucosa integrity but also activate airway epithelial cells [20]. Our results demonstrate impaired allergic lung inflammation and Th2 response with lower eosinophil influx and reduced IL-1*β*, IL-13 and IL-33 levels in the airways of TSLPR deficient mice. These findings were consistent with previous studies which demonstrate that TSLP may recruit eosinophils to sites of Th2 cytokine-associated inflammation by upregulating the common myeloid marker CD11b and the integrin *α*L*β*2 ligand ICAM-1 on eosinophils [15].

Dendritic cells are known to play a crucial role in allergic lung inflammation and are essential for T cell activation and Th2 cell differentiation and recruitment into the airways and trigger local Th2 cytokine production [37, 38]. We demonstrate reduced antigen uptake by myeloid TSLPR deficient DC and defective help for T cells measured by diminished T cell proliferation. Therefore, the defects in dendritic cell functions may affect Th2 cells differentiation, cytokines and chemokines productions in the lung of TSLPR-deficient mice.

While the role of TSLP on Th2 response is established, its effect on IL-17A and IL-22 cell response is novel. We has established a regulatory role for IL-17A and IL-22 in allergic asthma [32, 34, 35]. Our study in TSLPR deficient mice suggests that TSLPR signalling inhibits IL-17A expressing T cells and enhances the IL-22^+^ T cell response in the lung. These findings are novel and consistent with previous data demonstrating that IL-22 inhibits allergic lung inflammation by regulating IL-17A expression [34, 35]. 

IL-10 has broad immunosuppressive and anti-inflammatory actions relevant to the inhibition of asthma pathology. IL-10 has been found to be essential for effective suppression of allergic responses in the lung [39, 40]. IL-10 is a potent inhibitor of proinflammatory cytokine and acts on antigen-presenting cells to dampen T cell activation, including Th2 cells [41, 42]. Our results demonstrate increase IL-10 levels in the lung supernatant of TSLPR^−/−^ mice treated with HDM compared to WT mice. Therefore the data suggest TSLP modulates IL-10 and this might contribute to the inhibition of allergic inflammation in TSLPR^−/−^ mice.

These findings add to the complexity of the regulation of an allergic response where TSLPR signaling plays an important part [18, 36, 43, 44]. Furthermore, TSLPR dependent regulation of innate lymphoid cells producing IL-22 may contribute to the inflammatory response in the lung [45] and intestinal tract [46]. Therefore our data support the notion that TSLPR signaling in myeloid DC is required for T cell differentiation into Th2 and Th22 cells, which may control the IL-17A response. 

## Abbreviations

BAL: Bronchoalveolar lavage
EPO: Eosinophil peroxidase
HDM: House dust mite
OVA: Ovalbumin
TSLP: Thymic stromal lymphopoietin
PAS: Periodic acid Schiff.
